# Otolith Shape Variation Reveals a Preliminary and Novel Stock Structure Signal of Yellowtail Kingfish ( *Seriola lalandi* *lalandi*) in Aotearoa New Zealand

**DOI:** 10.1002/ece3.73810

**Published:** 2026-07-12

**Authors:** Carla H. Finn, Thomas C. Barnes, David Chagné, Maren Wellenreuther, Peter Ritchie

**Affiliations:** ^1^ Te Herenga Waka Victoria University of Wellington Aotearoa New Zealand; ^2^ Earth Sciences New Zealand Aotearoa New Zealand; ^3^ Institute of Marine and Antarctic Studies University of Tasmania Hobart Tasmania Australia; ^4^ Bioeconomy Science Institute, Nelson Aotearoa New Zealand; ^5^ University of Auckland Aotearoa New Zealand

**Keywords:** fisheries, otoliths, range expansion, Seriola, stock structure

## Abstract

Accurate stock delineation is essential for sustainable fisheries management, yet remains challenging in highly mobile marine species where conventional markers may lack resolution. Here, we present the first evaluation of sagittal otolith shape variation in Yellowtail kingfish (*Seriola lalandi lalandi*) across five sampling regions in Aotearoa New Zealand, using wavelet‐based contour descriptors and traditional shape indices to test for spatial structuring among sampling regions (*n* = 112 individuals), assessed via Canonical Analysis of Principal Coordinates (CAP) and permutation‐based PERMANOVA. Wavelet descriptors revealed statistically significant differences in otolith shape between Auckland west and all other sampled regions—a finding of particular relevance given the species' recent and ongoing southward range expansion, and the absence of previously documented stock‐level differentiation in this area. No significant differences were detected among southern regions. However, the proportion of variance explained was modest (CAP *R* ^2^ = 7.1%), indicating that the observed differences represent a preliminary signal of spatial structuring rather than confirmed stock boundaries. These results provide a baseline for future multi‐marker investigations to clarify the mechanisms driving the observed morphological differentiation and inform adaptive management of a range‐shifting species.

## Graphical abstract

Climate‐driven range expansion is reshaping marine species distributions and complicating stock assessment, including for Yellowtail Kingfish (*Seriola lalandi lalandi*) in Aotearoa New Zealand, which have recently expanded southward from a historically northern range. We analysed sagittal otolith shape from 112 individuals across five regions using wavelet‐based contour descriptors and traditional shape indices to assess spatial structure associated with this expansion. Otolith morphology differed significantly between the historic Auckland West population and all newly occupied regions, demonstrating its value for detecting stock structure and informing adaptive fisheries management under climate change.

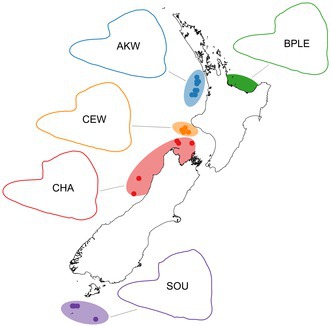

## 1 Introduction

The concept of stock is fundamental to modern fisheries management. A stock is defined as a reproductively or demographically distinct unit of fish, characterised by genetic, phenotypic, environmental, and/or harvest‐related traits (Carvalho and Hauser 1994; Ihssen et al. 1981); see also Waples and Gaggiotti (2006). Stocks are typically distinguished by limited gene flow, distinct growth and recruitment dynamics, and independent responses to exploitation (Cadrin et al. 2013). Correctly identifying stock boundaries is therefore a prerequisite for spatially relevant fisheries management: misalignment between management units and true population structure can lead to localised depletion and ultimately unsustainable harvest (Cadrin et al. 2013; Pita et al. 2016).

**FIGURE 1:**
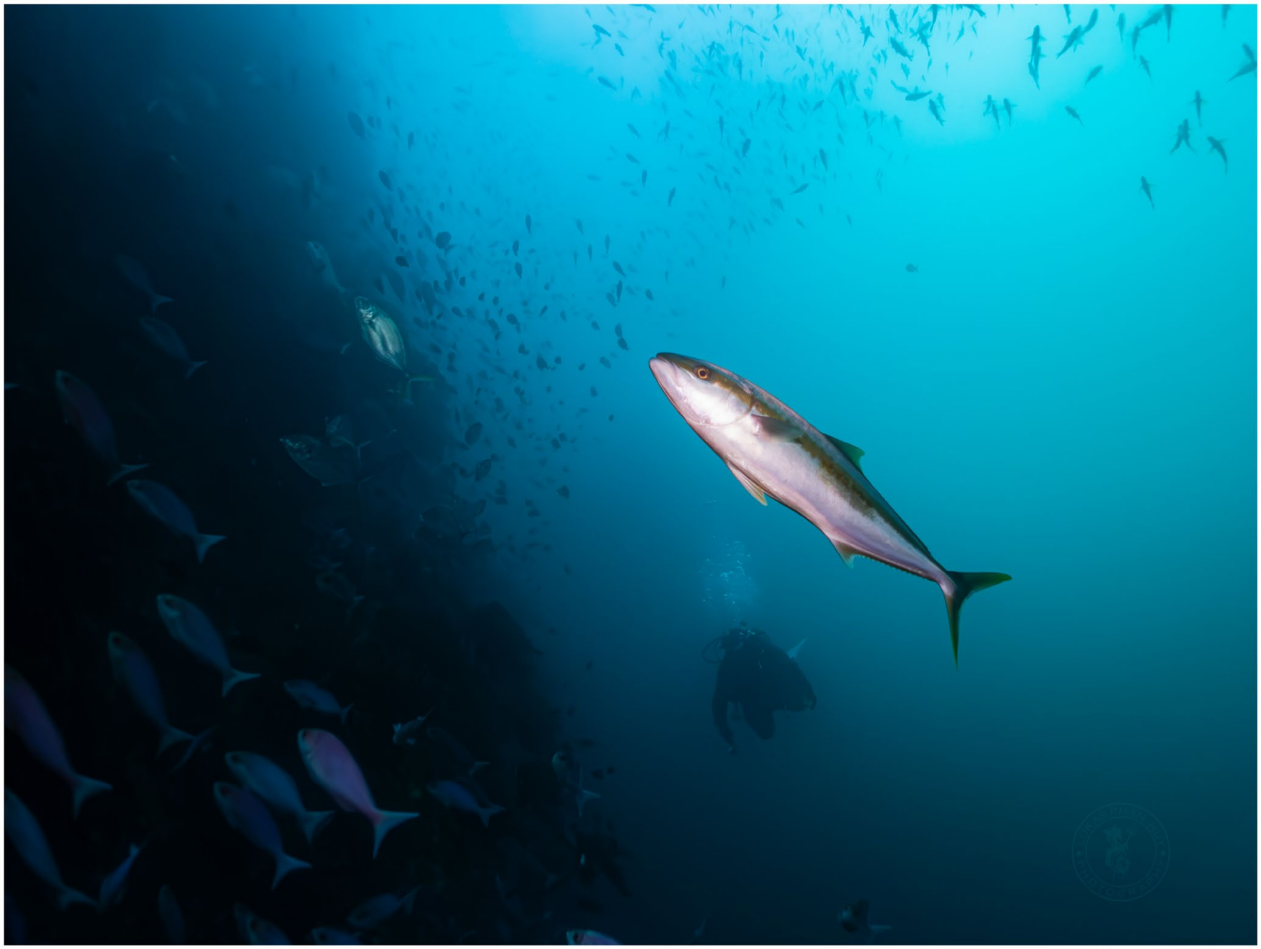
A Yellowtail kingfish from Northland, Aotearoa New Zealand. *Source:* Photograph © Lukas Phan‐huy, via iNaturalist NZ, licensed under CC BY‐NC 4.0. Observation date: 28 September 2024, Northern Arch, Poor Knights Islands, New Zealand.

In Aotearoa New Zealand, stock differentiation of Yellowtail kingfish (*Seriola lalandi lalandi*) (Figure 1) has become an increasingly pressing management question. Yellowtail kingfish are a warm‐temperate coastal‐pelagic species managed under the Quota Management System (QMS), which has been in place since 1986 (Cryer et al. 2016). Historically most abundant in the northern half of the North Island, the species has undergone a southward range expansion over the past decade, coinciding with rising sea surface temperatures and prompting renewed questions about whether current management boundaries remain appropriate (Champion et al. 2021; Fisheries New Zealand 2020). A key uncertainty is whether southern occurrences represent the establishment of new, demographically independent stock components, or whether they reflect temporary movements by individuals that remain connected to northern populations. This distinction has direct consequences for connectivity estimation, local exploitation risk, and the design of adaptive management strategies.

Previous stock structure investigations in this species within Aotearoa New Zealand have relied on meristic traits and parasite‐based markers. These approaches have suggested two potential stock boundaries in Aotearoa New Zealand, broadly segregated by the Tasman Current on the west coast and the East Auckland and East Cape Currents on the east coast (McKenzie 2014; Smith et al. 2004). However, these markers provide limited resolution of fine‐scale spatial structuring and carry methodological constraints—meristic counts are influenced by developmental conditions and are difficult to apply non‐invasively at scale, while parasite markers depend on host–parasite co‐occurrence patterns that may not map cleanly onto population boundaries (Cadrin et al. 2013). No study to date has examined otolith morphology in Yellowtail kingfish as a tool for investigating stock structure or connectivity across their New Zealand range.

In teleost fishes, three pairs of otoliths—sagitta, asteriscus, and lapillus—reside in the inner ear and play critical roles in balance and hearing (Das 1994). The sagittal otolith, the largest of the three, usually displays the most diversity in shape and size, and is the most widely used in ecological and stock structure studies (Campana and Casselman 1993; Popper et al. 2005). It is currently recognised that otolith shape is regulated by an interplay of genetic factors (Berg et al. 2018), and a wide range of ecological factors that alter otolith growth and therefore shape, including water temperature (Geladakis et al. 2022; Mahé et al. 2019), salinity (Clark et al. 2021), diet (Mille et al. 2016; Park et al. 2023), and water depth (Gauldie and Crampton 2002). Because shape divergence among populations tends to increase with geographic distance and genetic differentiation (Vignon and Morat 2010), otolith morphology serves as a useful integrative marker for identifying partially isolated stocks exposed to distinct environmental conditions, as reviewed in Nazir and Khan (2021) and Lishchenko and Jones (2021).

The development of digital imaging and mathematical contour descriptors—such as Fourier and wavelet transformations—has enhanced the precision and repeatability of otolith shape quantification (Libungan and Pálsson 2015; Park et al. 2023). These methods enable high‐resolution characterisation of the full otolith outline, facilitating the detection of subtle phenotypic divergence that is invisible to traditional scalar shape indices (Nazir and Khan 2021). Wavelet‐based approaches in particular offer the additional advantage of spatial localisation along the contour, enabling finer‐detail resolution for shape discrimination.

Here, we present the first evaluation of sagittal otolith shape variation in Yellowtail kingfish in Aotearoa New Zealand. Using wavelet‐based contour descriptors alongside traditional shape and size indices, we test whether otolith morphology can discriminate among individuals from five sampling regions, including both historically occupied northern areas and regions associated with the recent southward range expansion. Specifically, we ask: (i) does otolith shape vary significantly among sampling regions, and (ii) does any such variation align with the geographic distinction between historically occupied northern areas and regions associated with recent southward expansion? In doing so, we assess the utility of otolith morphology as a phenotypic marker for stock identification in this species and identify priorities for future multi‐marker investigations.

BOX 1.1 **Abbreviations used in this study.**

**Table:** 

*Sampling area abbreviations*
AKW, Auckland West
BPLE, Bay of Plenty
CEW, Central West
CHA, Challenger Plateau
SOU, Southland

## 2 Methods

A total of 112 Yellowtail kingfish individuals were collected from five sampling areas across Aotearoa New Zealand. The sampling locations are provided in Figure 1, and the numbers of individuals used in this study by year and sampling area are provided in Table 2. Abbreviations for the sampling areas are provided in Box 1.1.

**TABLE 1:** Numbers of individuals used in this study, by year and sampling area. Abbreviations for the sampling areas are provided in Box 1.1.

Year	Sampling area					
	CHA	AKW	BPLE	CEW	SOU	Total
2014		13	24			37
2019	20	13		6	1	40
2021		1		9		10
2023	13					13
2024					12	12
Total	33	27	24	15	13	112

Individuals were caught using commercial trawl targeting jack mackerel (sampling areas CHA, AKW, CEW and SOU), and angling (sampling area BPLE). Sagittal otoliths were extracted from each individual by onboard scientific observers. Due to the brittle nature of otoliths, either the left or right otolith was occasionally damaged during extraction. Paired left or right otoliths were successfully obtained from 29 individuals and were used for bilateral asymmetry analysis. Based on the results from this analysis, where a right otolith was unavailable, a mirror‐reflected image of the left otolith was substituted for analyses; this method is also demonstrated throughout the literature i.e., Aneesh Kumar et al. (2017), Ndjamba et al. (2022), and is similarly demonstrated with likewise results for the genus *Seriola* in Crandall et al. (2013).

### 2.1 Otolith Imaging

**FIGURE 2:**
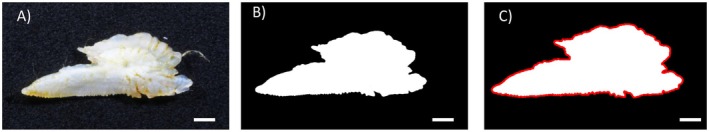
Demonstrating the otolith imaging process. (A) represents the raw otolith image, (B) represents the edited and centred otolith image, as edited using ImageJ (high‐contrast white denoting the otolith area), and (C) represents the outline function of the shapeR package (the red line denotes the perimeter). The scale bar in each image is 1 mm.

High‐resolution images of the otoliths were obtained using a Nikon stereo microscope (Nikon Instruments Inc., Tokyo, Japan) under reflected light coupled with NIS‐Elements software, version 5.3 (Nikon Instruments Inc., Tokyo, Japan). Otoliths were positioned on a dark microscope plate with the distal face oriented upward. All images were taken at a fixed magnification of 10× and saved in JPEG format. Before outline extraction, all images were processed in ImageJ version 1.54 g (Schneider et al. 2012) as follows: (1) debris was manually removed to produce clean, artefact‐free outlines suitable for automated detection, and any otoliths for which clean outlines could not be achieved were excluded from analysis; (2) contrasts were enhanced to achieve white otoliths against a black background; and (3) the otolith was centred within the frame. These steps were necessary to optimise outline detection by the ShapeR package (demonstrated in Figure 2).

### 2.2 Otolith Shape Outline Detection and Reconstruction

Outlines of otoliths were extracted using the detect.outline() function in the ShapeR package (version 1.0.1) (Libungan and Pálsson 2015) in RStudio (R version 4.4.1) (R Core Team 2019). A threshold value of 0.3 was applied to distinguish the otolith boundary. This value was determined by testing a range of threshold values (0.1–0.5) on a subset of images and selecting the value that most consistently produced complete, accurate outlines without artefacts, consistent with the approach described in Libungan and Pálsson (2015). Each detected outline was saved as a PNG image and manually inspected to check that the detected outline followed the contour of the otolith edge, as expected (e.g., see Figure 1). Following smoothing (100 iterations), shape descriptors were computed using both Wavelet and Elliptic Fourier (EF) transformations. All shape descriptors were standardised to fish total length (cm) using the stCoefs() function with Bonferroni adjustment enabled. No coefficients were excluded after standardisation for any analysis.

Reconstruction accuracy was assessed using the estimate.outline.reconstruction() function. Wavelet coefficients at level 9 reconstructed outlines with 100% accuracy (0% deviation), whereas EF harmonics achieved 98.8% at level 15 (Figure S2). As a result, all Wavelet coefficients were retained for subsequent shape analyses.

### 2.3 Otolith Contour Analyses

Wavelet coefficients were used to visualise otolith shape differences among sampling areas via Canonical Analysis of Principal Coordinates (CAP) (Anderson and Willis 2003). Ordination was performed using the capscale() function in the vegan package with sampling area as the constraining variable and Euclidean distances among standardised Wavelet coefficients as the response. The CAP model generates constrained axes, representing the portion of multivariate shape variation explained by sampling area. Significance of the constrained axes was evaluated using a permutation‐based ANOVA. Pairwise comparisons between sampling areas were tested using the PairwiseAdonis R package (version 0.4.1) (Arbizu 2020) with 999 permutations and Holm correction. To verify the assumption of homogeneity of multivariate dispersions, betadisper() and permutest() were applied from the vegan R package (version 2.6‐6.1) (Oksanen et al. 2016), followed by Tukey's HSD post hoc testing when applicable.

### 2.4 Otolith Indices

Otolith size indices—length (OL, mm), height (OH, mm), perimeter (OP, mm), and area (OA, mm^2^) were computed using ShapeR. Shape‐related indices were derived from these dimensions using formulas adapted from (Park et al. 2023), and included: form factor (FF), aspect ratio (AR), ellipticity (E), circularity (C), roundness (RO), and rectangularity (RE) (Table 2).

**TABLE 2:** Analytical expressions used to calculate otolith shape indices.

Index (abbreviation)	Equation
Circularity (C)	${\text{Perimeter}}^{2}/\text{Area}$
Rectangularity (RE)	$\text{Area}/\left(\text{Length}\times \text{Width}\right)$
Roundness (RO)	$4\times \text{Area}/\left(\pi \times {\text{Length}}^{2}\right)$
Aspect Ratio (AR)	Length/Width
Form Factor (FF)	$4\pi \times \text{Area}/{\text{Perimeter}}^{2}$
Ellipticity (E)	$\left(\text{Length}-\text{Width}\right)/\left(\text{Length}+\text{Width}\right)$

To account for allometric scaling with fish sizes (see Results), all indices were standardised using log–log regression against total fish length (Lleonart et al. 2000) with the equation:

(1) $$M_{s}=\exp\left(\log\left(M\right)+b\left[\log\left(\overset{⃐}{L}\right)-\log\left(L\right)\right]\right)$$

where M is the raw measurement (e.g., otolith area, shape index), $M_{s}$ is the standardised measurement, L is the total length of the individual fish, $\overset{⃐}{L}$ is the mean total length across all individuals, b is the allometric scaling coefficient, estimated as the slope from the regression of $\log\left(M\right)∼\log\left(L\right)$.

### 2.5 Preliminary Tests Used in This Study

Preliminary tests were conducted to assess the suitability of data before the sampling area differentiation testing.

#### 2.5.1 Prior Test 1: Left–Right Otolith Asymmetry

To assess bilateral asymmetry, standardised shape indices were compared between left and right otoliths for 29 individuals (58 otoliths total). Normality of paired differences was tested using the Shapiro–Wilk test. If normality was satisfied, a paired *t*‐test was conducted; otherwise, the Wilcoxon signed‐rank test was applied. Holm‐adjusted *p*‐values were calculated to account for multiple testing. There were no significant results between left and right otoliths (see Table S1).

To evaluate whether shape–length relationships differed by side, ANCOVA models were constructed for each shape index. A full model including an interaction term (side × length_cm) was compared against a reduced model using a likelihood ratio test. No interaction terms were statistically significant after correction (see Table S2).

Based on these tests, where the right otolith was not available for a given individual, a mirror‐imaged left otolith was used.

#### 2.5.2 Prior Tests 2–2.3: Temporal Comparisons Within Sampling Regions

To assess potential temporal variation in otolith morphology within sampling regions where multiple years of collection were available, standardised Wavelet coefficients were compared between years for AKW, CHA, and CEW independently. Comparisons were made between 2014 (n=13) and 2019 (n=13) for AKW; 2019 (n=20) and 2023 (n=13) for CHA; and 2019 (n=6) and 2021 (n=9) for CEW. Otolith shape ordination was performed as described in 2.3 Otolith contour analyses for each comparison independently. It is noted that the CEW 2019 and 2021 group comprises only six and nine individuals, respectively, which limits the statistical power of the CEW comparison.

No significant differences in shape profiles were detected between years in any region. For AKW (preliminary test 2.1): PERMANOVA F=0.627, $R^{2}=0.026$, p=0.781; PERMDISP F=0.007, p=0.943. For CHA (preliminary test 2.2): PERMANOVA F=0.920, $R^{2}=0.029$, p=0.504; PERMDISP F=0.035, p=0.856. For CEW (prelimary test 2.3): PERMANOVA F=0.766, $R^{2}=0.056$, p=0.635; PERMDISP F=1.656, p=0.230. Consequently, samples from all years were pooled within each sampling area for subsequent analyses (see Figure S1a–c).

### 2.6 Simulation‐Based Power Analysis

To evaluate the adequacy of sample sizes relative to the observed effect size, a simulation‐based power analysis was conducted by bootstrapping from the observed standardised Wavelet coefficient distributions across a range of equal sample sizes per group (n=10–100; 999 iterations; α=0.05), with the proportion of iterations yielding a significant PERMANOVA result recorded as the estimated power at each sample size (Kelly et al. 2015) (Figure S3).

### 2.7 Stock‐Level Differences

#### 2.7.1 Otolith Contour Analysis

To assess spatial variation in otolith shape among sampling regions, we applied the same wavelet descriptor procedure described in Section 2.3. Due to a significant violation of dispersion homogeneity in the BPLE sampling area, BPLE was excluded from PERMANOVA testing (see Section 3.3.1). It is additionally noted that BPLE individuals were the only group collected by angling rather than commercial trawl, which may have introduced compositional or habitat‐related sampling differences relative to other areas; this is discussed further in Section 4.

To evaluate the discriminatory power of otolith shape for assigning individuals to sampling area of origin, we performed a Linear Discriminant Analysis (LDA) using standardised Wavelet coefficients as predictors. The LDA was implemented using the MASS package (version 7.3–60) (Kemp 2003) with leave‐one‐out cross‐validation (LOOCV).

#### 2.7.2 Otolith Shape Indices Analysis

To assess shape and size indices differences, we applied the same shape indices procedure described in Section 2.4. Each otolith size and shape index were assessed for normality using the Shapiro–Wilk test. Variables that violated the assumption of normality (p<0.05) were subsequently categorised for non‐parametric testing. Homogeneity of variance across sampling areas was assessed for each index using Levene's test (as implemented in the rstatix R package, version 0.7.2, Kassambara 2021).

Based on these assumptions, the following analytical approach was used:

- For indices that met both normality and homogeneity assumptions, a one‐way ANOVA was conducted, followed by Tukey's Honest Significant Difference (HSD) test for post hoc pairwise comparisons.
- For indices that were normally distributed but violated the homogeneity of variance assumption, Welch's ANOVA was used, followed by Games–Howell post hoc tests.
- For indices that failed normality, a Kruskal–Wallis test was performed, followed by Dunn's test for post hoc pairwise comparisons with Holm‐adjusted p‐values (as implemented in the rstatix R package).

#### 2.7.3 Packages and Scripts in This Study

All statistical tests were as implemented either in base packages belonging to R version 4.4.1 (base R) (R Core Team 2019), or otherwise in packages specified in‐text. Other packages used for purpose of data wrangling are: dplyr (version 1.1.4) (Hadley Wickham et al. 2020) tidyr (version 1.3.1) (Wickham et al. 2024) ggplot2 (version 3.5.2) (Wickham 2011).

## 3 Results

**FIGURE 3:**
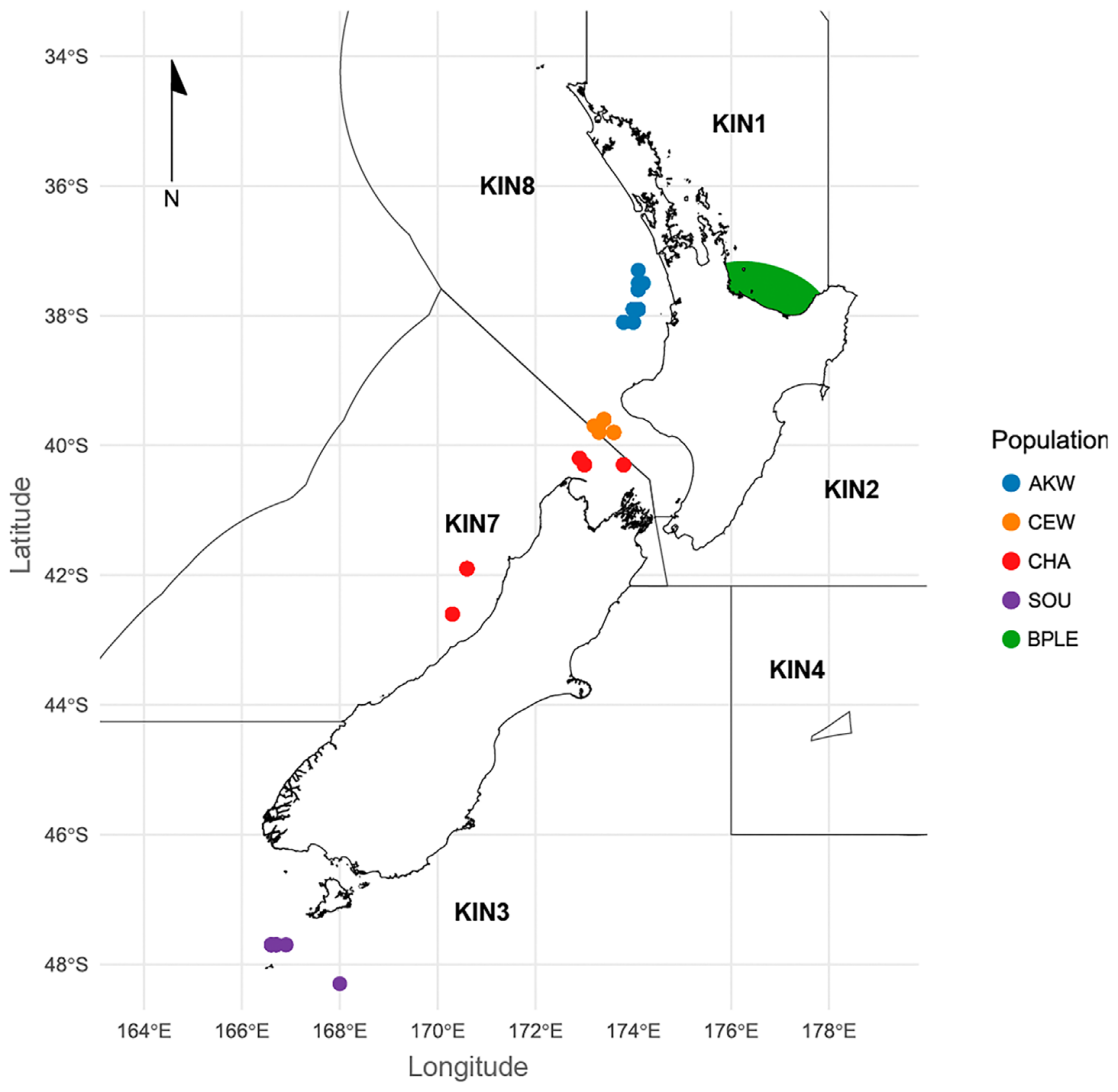
Map showing the locations of the individual Kingfish per sampling area. Note that the precise location of BPLE cannot be released due to confidentiality reasons; therefore, the general sampling area has been provided. The map has been superimposed onto the Yellowtail kingfish Fishery Management areas for NZ, as provided by the Ministry of Primary Industries (MPI 2002). Abbreviations for the sampling areas are provided in Box 1.1. Sampling area counts are provided in Table 1.

A total of 112 Yellowtail kingfish individuals, sampled across five sampling areas, were included in the otolith analysis. These sampling sites span a longitudinal gradient across Aotearoa New Zealand (Figure 3; abbreviations for the sampling areas provided in Box 1.1). Sample composition was as follows: 27 BPLE individuals (75–123.5 cm), 15 CEW individuals (71–90 cm), 33 CHA individuals (67–112 cm), 13 SOU individuals (90–109 cm), and 27 AKW individuals (67–87 cm).

### 3.1 Power Analysis

To evaluate the adequacy of our sample sizes relative to the observed effect size, we conducted a simulation‐based power analysis. Results indicated that 80% statistical power is achieved at approximately n=15 individuals per group, given the effect size observed in this study (PERMANOVA $R^{2}=0.071$; Figure S3). Three of the four groups analysed—CEW (n=15), AKW (n=27), and CHA (n=33) – meet or exceed this threshold, achieving estimated power of 81%, 100%, and 100%, respectively. The Southland group (SOU, n=13) falls marginally below the 80% threshold at an estimated power of 44%, representing a limitation that is acknowledged accordingly. These results suggest that the observed sample sizes are broadly adequate for the effect size present in the data, with the exception of SOU, where conclusions should be treated with additional caution.

### 3.2 Otolith Indices

Descriptive statistics for otolith size and shape indices across the five sampling locations are presented in Table 3. Neither parametric nor non‐parametric tests revealed significant differences in any single size or shape index across sampling areas, suggesting that no individual metric independently captures the multivariate shape variation observed among groups.

**TABLE 3:** Summary of otolith size and shape indices across sampling locations. Abbreviations for the sampling areas are provided in Box 1.1. Values are reported as Min–Max (mean ± SD).

Standardised variables	Min–Max (Mean ± SD)				
	AKW	BPLE	CEW	CHA	SOU
Aspect ratio	2.40–3.27 (2.76 ± 0.22)	2.19–3.27 (2.70 ± 0.36)	2.37–3.23 (2.76 ± 0.26)	2.22–3.21 (2.72 ± 0.23)	2.18–3.33 (2.73 ± 0.36)
Circularity	27.34–38.19 (33.52 ± 2.78)	29.55–44.53 (35.46 ± 3.43)	28.20–40.02 (31.73 ± 3.11)	25.44–39.86 (33.56 ± 3.15)	26.91–40.95 (32.92 ± 3.54)
Ellipticity	0.41–0.53 (0.45 ± 0.03)	0.37–0.46 (0.41 ± 0.03)	0.41–0.52 (0.45 ± 0.03)	0.38–0.52 (0.45 ± 0.04)	0.35–0.53 (0.41 ± 0.05)
Form factor	0.33–0.46 (0.39 ± 0.03)	0.28–0.57 (0.42 ± 0.26)	0.31–0.44 (0.38 ± 0.03)	0.32–0.49 (0.41 ± 0.03)	0.31–0.43 (0.38 ± 0.03)
Rectangularity	0.45–0.53 (0.48 ± 0.02)	0.51–0.69 (0.60 ± 0.04)	0.45–0.58 (0.52 ± 0.03)	0.36–0.63 (0.52 ± 0.06)	0.52–0.63 (0.56 ± 0.04)
Roundness	0.21–0.31 (0.27 ± 0.04)	0.22–0.37 (0.29 ± 0.04)	0.23–0.30 (0.27 ± 0.02)	0.23–0.38 (0.28 ± 0.03)	0.22–0.34 (0.26 ± 0.03)
Otolith area	11.51–21.63 (13.97 ± 2.21)	11.40–17.57 (14.55 ± 1.80)	11.46–15.19 (13.19 ± 0.99)	10.66–17.46 (12.47 ± 1.89)	10.68–15.79 (13.66 ± 1.41)
Otolith length	7.14–8.81 (8.69 ± 0.50)	6.48–10.01 (8.20 ± 0.86)	7.43–8.88 (7.93 ± 0.41)	6.91–9.26 (8.15 ± 0.57)	6.48–9.10 (8.07 ± 0.57)

### 3.3 Average Shape Contours

**FIGURE 4:**
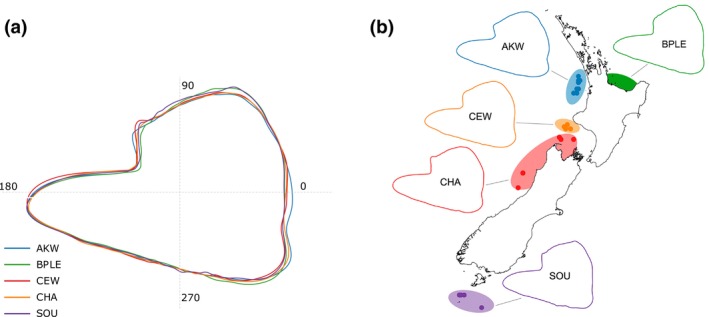
Average otolith contours, as generated using the shapeR package, which utilises wavelet coefficients. (a) Average otolith contour, with angles given in degrees. Contours are overlaid for comparison. (b) Average otolith contour for each sampling area. Points represent individuals. Note that the precise location of BPLE cannot be released due to confidentiality reasons; therefore, the general sampling area has been provided. Coloured by sampling area. Abbreviations for the sampling areas are provided in Box 1.1.

Average otolith outlines based on wavelet coefficients (0°–360°) showed subtle differences in mean shape among sampling areas, particularly near 90° and 180°, corresponding to the antirostrum. CEW individuals exhibited a more rounded and elevated antirostrum, whereas BPLE displayed a flatter, less inflected contour. In contrast, AKW individuals had a sharply inflected and dorsally depressed antirostrum, positioned lower than other groups (Figure 4). These differences were diffuse across the contour rather than concentrated at any single landmark, consistent with the absence of significant differences in traditional shape indices and underscoring the advantage of whole‐contour wavelet analysis for detecting subtle morphological variation.

#### 3.3.1 Shape Contour Analysis

Wavelet coefficients were analysed using canonical analysis of principal coordinates (CAP). Prior to permutation tests, we assessed the homogeneity of multivariate dispersions using betadisper() followed by permutest(), which indicated significant differences among groups (p=0.002). Post hoc Tukey's HSD tests revealed that the BPLE sampling area exhibited significantly greater dispersion than both AKW (p=0.001) and CEW (p=0.038) (see Table S3), thereby violating the assumption of dispersion homogeneity required for valid PERMANOVA. It is noted that BPLE individuals were collected using angling rather than the commercial trawl method used for all other sampling areas; while this gear difference may have introduced some compositional bias, the elevated within‐group dispersion is also consistent with BPLE's previously described role as a stock boundary zone for this species (McKenzie 2014; Smith et al. 2004). Consequently, BPLE was excluded from subsequent PERMANOVA testing, though its potential biological significance is discussed further in Section 4.

After excluding BPLE, homogeneity of dispersion among remaining groups was no longer significant (p=0.075).

**FIGURE 5:**
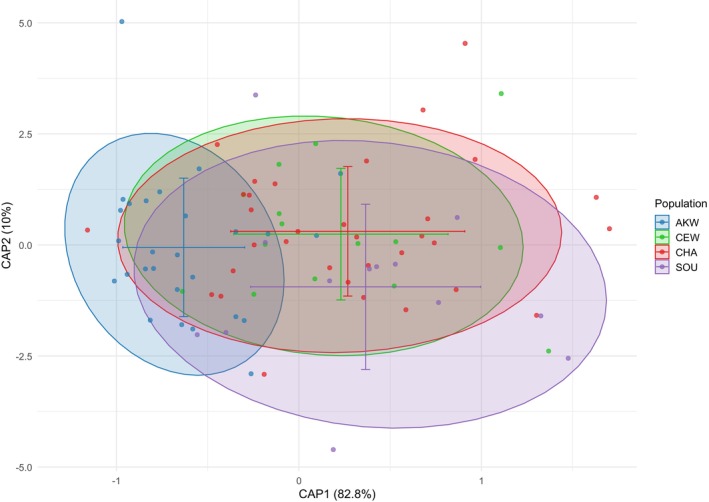
Canonical analysis of principal coordinates (CAP) using Euclidean distances of standardised wavelet coefficients, shown for four Yellowtail kingfish *S. lalandi lalandi* sampling areas in Aotearoa New Zealand. BPLE was excluded due to high within‐group dispersion. Points represent individuals; crosses indicate group centroids with bars showing ±1 SD. Ellipses denote 95% confidence intervals under multivariate normality.

The CAP model indicated a statistically significant effect of sampling area on otolith shape (Permutation test: F=2.13, p=0.001), explaining 7.1% of the total variation in shape space. While statistically significant, this proportion of explained variance is modest, suggesting that otolith shape captures a limited but detectable signal of spatial structuring among sampling areas. Sequential term testing confirmed sampling area as a significant explanatory variable (p=0.002). Axis‐wise testing revealed that only the first canonical axis (CAP1) contributed significantly to group separation (p=0.001), while CAP2 and CAP3 were non‐significant. Thus, shape variation among locations was primarily captured along CAP1 (Figure 5).

Pairwise PERMANOVA comparisons (Table 4) revealed significant shape differences between AKW and each of CEW, CHA, and SOU (adjusted p=0.006). No significant differences were observed among CEW, CHA, or SOU (adjusted p=1.000), indicating that AKW is the primary driver of inter‐sampling area shape divergence. The pairwise $R^{2}$ values for AKW comparisons ranged from 0.072 to 0.080, indicating that while the differences are statistically significant, they account for a small proportion of total shape variation, and should be interpreted as evidence of possible rather than confirmed demographic differentiation.

**TABLE 4:** Pairwise PERMANOVA comparisons of wavelet‐based otolith shape among sampling areas, showing *F*‐statistics, *R* ^2^, and raw and Holm‐adjusted *p*‐values. Asterisks indicate statistically significant differences (*p* < 0.05). Abbreviations for the sampling areas are provided in Box 1.1.

Comparison	*F*‐value	$R^{2}$	Raw *p*	Holm‐adjusted *p*	Significant
AKW vs. CEW	3.15	0.073	0.001	0.006	*
AKW vs. CHA	4.50	0.072	0.001	0.006	*
AKW vs. SOU	3.30	0.080	0.001	0.006	*
CEW vs. CHA	0.46	0.010	0.941	1.000	
CEW vs. SOU	0.53	0.020	0.871	1.000	
CHA vs. SOU	0.58	0.013	0.824	1.000	

**FIGURE 6:**
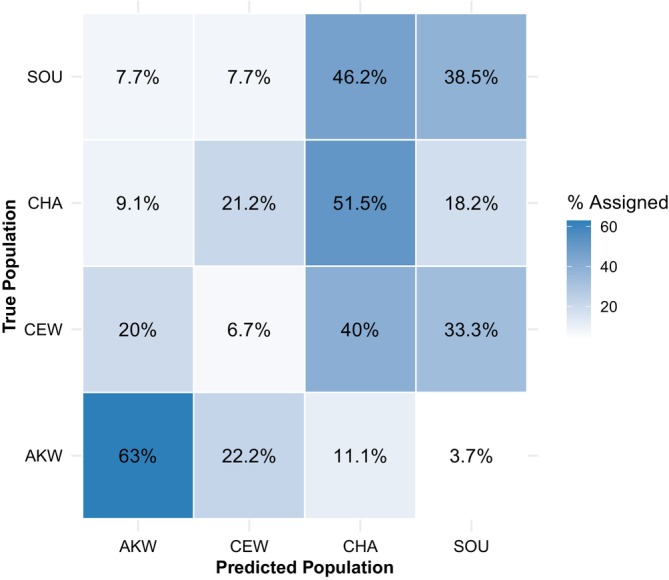
LOOCV Confusion Matrix—Otolith Shape Classification based on standardised wavelet coefficients (LDA model). Abbreviations for the sampling areas are provided in Box 1.1.

Classification using LOOCV (Figure 6) resulted in an overall correct assignment rate of 45.5%, which reflects the substantial overlap among sampling areas evident in the CAP ordination. The highest classification success was observed for AKW individuals (63%), consistent with the separation of this group along CAP1. Classification performance was markedly lower for CEW and SOU, with only 20% and 7.7% of individuals correctly assigned, respectively, suggesting that otolith shape alone provides insufficient resolution for routine stock assignment in these regions. These results indicate that otolith shape contains a detectable population‐level signal, but that its discriminatory power is limited and best interpreted as a preliminary indication of spatial structuring rather than a definitive basis for stock delineation.

## 4 Discussion

This study presents the first application of otolith shape analysis to assess spatial structuring in Yellowtail kingfish (*Seriola lalandi lalandi*) across Aotearoa New Zealand. Because otolith shape integrates both genetic and ecological (e.g., temperature, diet, habitat use) influences accumulated over the lifetime of an individual, it has the potential to retain signatures of divergent life histories (Berg et al. 2018; Vignon and Morat 2010). Using wavelet descriptors, we detected statistically significant differences in otolith shape between the Auckland west (AKW) sampling area and all other regions. However, the proportion of variation explained (CAP *R* ^2^ = 7.1%) and overall classification success (45.5%) are both modest, and we caution that these results are better interpreted as preliminary evidence of possible spatial structuring than as confirmation of established stock boundaries. The observed patterns are consistent with the possibility that southern sampling areas contain fish that are less demographically established than those in AKW, but further sampling and complementary markers will be required to substantiate this interpretation.

### 4.1 Otolith Shape Indices Versus Wavelet Descriptors

Traditional shape indices—including form factor, circularity, ellipticity, aspect ratio, roundness, and rectangularity—revealed no significant differences among any sampling area (Table 3). This outcome warrants discussion, as it stands in contrast to the significant multivariate differences detected by wavelet descriptors. Shape indices summarise the overall geometry of the otolith in a small number of scalar values, and by doing so, they discard information about localised features of the contour. Wavelet descriptors, by contrast, decompose the otolith outline across multiple spatial scales and angular positions, enabling the detection of subtle, localised morphological differences that are otherwise undetectable by summary indices (Libungan and Pálsson 2015; Parisi‐Baradad et al. 2005). In the present study, shape variation in AKW appears to be diffuse across the otolith contour rather than concentrated in any single geometric property, which explains why indices fail to capture it. This result underscores both a known limitation of index‐based approaches and a key advantage of whole‐contour wavelet analysis for detecting subtle inter‐population divergence (Lishchenko and Jones 2021; Nazir and Khan 2021).

It is worth noting that wavelet descriptors and elliptic Fourier descriptors (EFDs) differ in how they characterise contours: EFDs decompose shape globally by frequency, whereas wavelets additionally preserve spatial localisation, enabling identification of where along the contour differences occur (Libungan and Pálsson 2015; Parisi‐Baradad et al. 2005). In practice, EFD's representation of the outline is limited by the number of harmonics used, which can result in underrepresentation of fine contour detail—a particular concern where populations differ in subtle rather than gross morphological features, as is the case in closely related populations or species (Lishchenko and Jones 2021; Reig‐Bolaño et al. 2010). When the efficiency of EFD's, Fast fourier transform, and wavelet transform was directly compared for species discrimination across 11 *Lutjanus* spp., wavelet analysis produced the best results (Sadighzadeh et al. 2012). The superior reconstruction accuracy achieved by wavelets in the present study (100% at level 9, vs. 98.8% for EFDs at level 15) is consistent with this, and further supports the suitability of wavelet‐based contour analysis for detecting the subtle morphological differentiation observed here.

### 4.2 Spatial Structuring in Yellowtail Kingfish

The AKW sampling area exhibited a distinctly different otolith contour shape relative to all other regions, with significant pairwise differences confirmed against CEW, CHA, and SOU (adjusted p=0.006). This is notable given that population differentiation in this region has not previously been reported for this species. While the observed divergence may indicate some degree of ecological or demographic separation within the northerly historic range, we note that the effect sizes are small (*R* ^2^ =0.072–0.080), classification success for AKW is moderate at 63%, and the present sample sizes—particularly for SOU (n=13, estimated power 44%)—limit the confidence with which differences among groups can be detected or excluded.

In pelagic species such as Yellowtail kingfish, morphological divergence is rarely expected due to high dispersal potential, large effective population sizes, and low site fidelity. However, whole‐contour shape analysis has revealed cryptic spatial structuring in other pelagic fish species with similarly high dispersal potential, including *Sardina pilchardus* (Neves et al. 2023), *Clupea harengus* (Libungan et al. 2015), and *Trachurus trachurus* (Vasconcelos et al. 2025).

An important caveat is that the observed differences may reflect phenotypic plasticity driven by environmental gradients—such as differences in temperature, salinity, or diet across the sampled range—rather than demographic or genetic stock structure (Clark et al. 2021; Mahé et al. 2019; Mille et al. 2016). Without supporting genetic or environmental covariate data, it is not possible to distinguish between these explanations. Fish age represents a further unresolved source of variation: ontogenetic effects on calcification rate are known to influence otolith shape independently of population origin (Campana and Casselman 1993; Lishchenko and Jones 2021). Allometric growth changes in shape associated with age and growth were mitigated in the present study by standardising all shape descriptors to fish total length, following established recommendations (Lishchenko and Jones 2021; Lleonart et al. 2000); however, length standardisation does not fully account for age where individual growth rates vary, and direct age estimation remains preferable where feasible. Age determination in *S. lalandi* is complicated by difficulties in reading the first growth ring in wild‐caught individuals (Ndjamba et al. 2022), and maximum age estimates for this species vary widely across localities and methods, ranging from 7 to 21 years (McKenzie 2014; Ndjamba et al. 2022). Future work could address this limitation through otolith weight‐based ageing, which has been demonstrated as a practical alternative to annuli counting for this species in Chile (Ndjamba et al. 2022); conducting similar research for the New Zealand locale would enable age‐structured reanalysis of the shape variation reported here.

It is further acknowledged that fish sex was not available for individuals in this study; however, sex‐related differences in otolith shape are generally considered minor relative to environmental and ontogenetic effects in teleosts, and are frequently found to be non‐significant in comparable studies (Lishchenko and Jones 2021).

The relative homogeneity among CEW, CHA, and SOU—and the difficulty of correctly classifying individuals from these regions (20%, 7.7% and 9.1% respectively)—is consistent with the interpretation that fish in these southern areas may represent a relatively recently arrived, demographically mixed expansion front rather than long‐established independent stocks. Otolith shape analysis compares favourably to molecular approaches in several practical respects: it is relatively inexpensive, does not require specialised genetic laboratory infrastructure, and integrates environmental as well as genetic influences over the lifetime of the fish (Cadrin et al. 2013; Nazir and Khan 2021). However, it is less able to unambiguously assign observed variation to genetic versus environmental causes, and its discriminatory power in high‐connectivity species such as Yellowtail kingfish may be inherently limited (Campana and Casselman 1993). A multi‐marker approach combining otolith contour analysis with genomic and stable isotope data would provide substantially greater resolution for stock delineation, as demonstrated in other species (Longmore et al. 2010; Neves et al. 2019; Randon et al. 2020; Pita et al. 2016). Such integrative analyses are recommended as a priority for future work on this species.

### 4.3 Heterogeneity Within the Bay of Plenty Group

Multivariate dispersion within the Heterogeneity Within the Bay of Plenty Group (BPLE) sampling group was significantly higher than in AKW or CEW, violating the assumption of homogeneity required for PERMANOVA and necessitating its exclusion from comparative analyses. It is noted that BPLE individuals were the only group collected by angling rather than commercial trawl, which may have introduced some compositional or habitat‐related sampling bias relative to the other areas. This gear difference should be considered when interpreting the BPLE result, and future sampling of this region using consistent methods would help to clarify whether the elevated dispersion reflects true biological heterogeneity or a methodological artefact.

Notwithstanding this uncertainty, the elevated within‐group variation is also consistent with prior evidence that BPLE represents a biological transition zone for Yellowtail kingfish populations. Significant meristic variation between eastern and western populations has been reported, and parasite‐based markers have suggested a potential stock boundary in this region (McKenzie 2014; Smith et al. 2004). These findings raise the possibility that the BPLE sample includes individuals from distinct ecological or demographic backgrounds, and this region warrants targeted follow‐up research with larger and methodologically consistent sampling.

## 5 Conclusions

This study provides the first otolith shape data for Yellowtail kingfish in Aotearoa New Zealand, and demonstrates that wavelet‐based whole‐contour analysis is capable of detecting subtle morphological differences among sampling areas. The distinctiveness of AKW otolith shapes relative to southern sampling areas is an encouraging preliminary signal, and is consistent with the hypothesis that AKW fish may be ecologically or demographically differentiated from individuals associated with the southward range expansion. The management implications of resolving this question are substantial: if fish in southern regions remain demographically connected to AKW, managing them as independent units risks underestimating stock size, whereas confirmed morphological divergence would signal the need to revise current management boundaries. Future work should prioritise increased sampling across all regions—particularly SOU—and the integration of otolith shape with genomic and isotopic markers to resolve the mechanisms and persistence of the observed morphological differentiation.

## Author Contributions

**Carla H. Finn:** conceptualization (equal), data curation (lead), formal analysis (lead), investigation (lead), methodology (lead), visualization (lead), writing – original draft (lead), writing – review and editing (lead). **Thomas C. Barnes:** conceptualization (equal), data curation (supporting), investigation (supporting), resources (lead), supervision (equal). **David Chagné:** supervision (supporting), writing – review and editing (supporting). **Maren Wellenreuther:** supervision (supporting), writing – review and editing (supporting). **Peter Ritchie:** conceptualization (supporting), supervision (equal), writing – review and editing (supporting).

## Funding

This work was supported by Victoria University of Wellington.

## Conflicts of Interest

The authors declare no conflicts of interest.

## Supporting information


**Figure S1:** CAP ordination plots for temporal comparisons within sampling regions (Prior Tests 2.1–2.3). Ellipses indicate 95% confidence regions based on multivariate normality. Crosshairs indicate group centroids ±1 SD. Substantial overlap between years in all three regions supports pooling of samples across years for subsequent analyses.
**Figure S2:** Mean deviation from the original outline (%) as a function of the number of Fourier harmonics (left), and the number of wavelet levels (right), used in shape reconstruction.
**Figure S3:** Simulation‐based power analysis for PERMANOVA on standardised wavelet coefficients (four groups: AKW, CEW, CHA, SOU; BPLE excluded; α=0.05; 999 iterations). Power was estimated by bootstrapping from the observed wavelet coefficient distributions across a range of equal sample sizes per group. The red dashed line indicates the 80% power threshold. Dotted vertical lines indicate the actual sample sizes of each sampling area. Three of the four groups—CEW (n=15), AKW (n=27), and CHA (n=33)—meet or exceed the 80% threshold. SOU (n=13) falls marginally below, representing an acknowledged limitation.
**Table S1:** Comparison of left and right otolith shape indices. Normality assessed using the Shapiro–Wilk test. Holm‐adjusted p‐values indicate no significant asymmetry.
**Table S2:** ANCOVA interaction effects of side and fish length on otolith shape indices. Holm‐adjusted p‐values indicate no significant interaction.
**Table S3:** Pairwise Tukey's HSD comparisons of multivariate dispersion (distance to group centroid). Values show difference in dispersion (Diff) and Holm‐adjusted p‐values. Significant values indicate heterogeneity in within‐group variance.

## Data Availability

All data for repeating this analysis, including the code used, can be found at: https://doi.org/10.5061/dryad.gmsbcc335.

## References

[ece373810-bib-0001] Anderson, M. J. , and T. J. Willis . 2003. “Canonical Analysis of Principal Coordinates: A Useful Method of Constrained Ordination for Ecology.” Ecology 84, no. 2: 511–525. 10.1890/0012-9658(2003)084[0511:CAOPCA]2.0.CO;2.

[ece373810-bib-0002] Aneesh Kumar, K. V. , R. Nikki , K. Oxona , M. Hashim , and M. Sudhakar . 2017. “Relationships Between Fish and Otolith Size of Nine Deep‐Sea Fishes From the Andaman and Nicobar Waters, North Indian Ocean.” Journal of Applied Ichthyology 33, no. 6: 1187–1195. 10.1111/jai.13467.

[ece373810-bib-0003] Arbizu, P. M. 2020. “pairwiseAdonis: Pairwise Multilevel Comparison Using Adonis.”

[ece373810-bib-0004] Berg, F. , O. W. Almeland , J. Skadal , A. Slotte , L. Andersson , and A. Folkvord . 2018. “Genetic Factors Have a Major Effect on Growth, Number of Vertebrae and Otolith Shape in Atlantic Herring ( *Clupea harengus* ).” PLoS One 13, no. 1: e0190995. 10.1371/journal.pone.0190995. 29324892 PMC5764352

[ece373810-bib-0005] Cadrin, S. X. , L. A. Kerr , and S. Mariani . 2013. “Stock Identification Methods: An Overview.” In Stock Identification Methods: Applications in Fishery Science: Second Edition. Academic Press. 10.1016/B978-0-12-397003-9.00001-1.

[ece373810-bib-0006] Campana, S. E. , and J. M. Casselman . 1993. “Stock Discrimination Using Otolith Shape Analysis.” Canadian Journal of Fisheries and Aquatic Sciences 50, no. 5: 1062–1083. 10.1139/f93-123.

[ece373810-bib-0007] Carvalho, G. R. , and L. Hauser . 1994. “Molecular Genetics and the Stock Concept in Fisheries.” Reviews in Fish Biology and Fisheries 4, no. 3: 326–350. 10.1007/BF00042908.

[ece373810-bib-0008] Champion, C. , S. Brodie , and M. A. Coleman . 2021. “Climate‐Driven Range Shifts Are Rapid Yet Variable Among Recreationally Important Coastal‐Pelagic Fishes.” Frontiers in Marine Science 8. 10.3389/fmars.2021.622299.

[ece373810-bib-0010] Clark, F. J. K. , C. S. da Silva Lima , and A. L. M. Pessanha . 2021. “Otolith Shape Analysis of the Brazilian Silverside in Two Northeastern Brazilian Estuaries With Distinct Salinity Ranges.” Fisheries Research 243: 106094. 10.1016/j.fishres.2021.106094.

[ece373810-bib-0011] Crandall, C. A. C. , D. C. Parkyn , and D. J. Murie . 2013. Regional Stock Structure of Greater Amberjack in the Southeastern United States Using Otolith Shape Analysis (Tech. Rep. No. SEDAR33‐DW25). SEDAR.

[ece373810-bib-0012] Cryer, M. , P. M. Mace , and K. J. Sullivan . 2016. “New Zealand's Ecosystem Approach to Fisheries Management.” Fisheries Oceanography 25: 57–70. 10.1111/fog.12088.

[ece373810-bib-0014] Das, M. 1994. “Age Determination and Longevity in Fishes.” Gerontology 40, no. 2–4: 70–96. 10.1159/000213580. 7926859

[ece373810-bib-0015] Fisheries New Zealand . 2020. “Proposal to Vary the Total Allowable Catch and Total Allowable Commercial Catch for Kingfish (KIN 3).” https://www.nzsportfishing.co.nz/wp‐content/uploads/2020/08/KIN‐proposal‐FNZ‐May20.pdf.

[ece373810-bib-0016] Gauldie, R. W. , and J. S. Crampton . 2002. “An Eco‐Morphological Explanation of Individual Variability in the Shape of the Fish Otolith: Comparison of the Otolith of *Hoplostethus atlanticus* With Other Species by Depth.” Journal of Fish Biology 60, no. 5: 1204–1221. 10.1006/jfbi.2002.1938.

[ece373810-bib-0017] Geladakis, G. , C. Kourkouta , S. Somarakis , and G. Koumoundouros . 2022. “Developmental Temperature Shapes the Otolith Morphology of Metamorphosing and Juvenile Gilthead Seabream ( *Sparus aurata* Linnaeus, 1758).” Fishes 7, no. 2: 82. 10.3390/fishes7020082.

[ece373810-bib-0018] Ihssen, P. E. , H. E. Booke , J. M. Casselman , J. M. McGlade , N. R. Payne , and F. M. Utter . 1981. “Stock Identification: Materials and Methods.” Canadian Journal of Fisheries and Aquatic Sciences 38, no. 12: 1838–1855. 10.1139/f81-230.

[ece373810-bib-0019] Kassambara, A. 2021. “Pipe‐Friendly Framework for Basic Statistical Tests [R Package ‘rstatix’ Version 0.7. 0].”

[ece373810-bib-0020] Kelly, B. J. , R. Gross , K. Bittinger , et al. 2015. “Power and Sample‐Size Estimation for Microbiome Studies Using Pairwise Distances and PERMANOVA.” Bioinformatics 31, no. 15: 2461–2468. 10.1093/bioinformatics/btv183. 25819674 PMC4514928

[ece373810-bib-0021] Kemp, F. 2003. “Modern Applied Statistics With S.” Journal of the Royal Statistical Society: Series D (The Statistician) 52, no. 4. 10.1046/j.1467-9884.2003.t01-19-00383{∖}22.x.

[ece373810-bib-0022] Libungan, L. A. , G. J. Óskarsson , A. Slotte , J. A. Jacobsen , and S. Pálsson . 2015. “Otolith Shape: A Population Marker for Atlantic Herring *Clupea harengus* .” Journal of Fish Biology 86, no. 4: 1377–1395. 10.1111/jfb.12647. 25846860

[ece373810-bib-0023] Libungan, L. A. , and S. Pálsson . 2015. “ShapeR: An R Package to Study Otolith Shape Variation Among Fish Populations.” PLoS One 10, no. 3: e0121102. 10.1371/journal.pone.0121102. 25803855 PMC4372608

[ece373810-bib-0024] Lishchenko, F. , and J. B. Jones . 2021. “Application of Shape Analyses to Recording Structures of Marine Organisms for Stock Discrimination and Taxonomic Purposes.” Frontiers in Marine Science 8: 667183. 10.3389/fmars.2021.667183.

[ece373810-bib-0025] Lleonart, J. , J. Salat , and G. J. Torres . 2000. “Removing Allometric Effects of Body Size in Morphological Analysis.” Journal of Theoretical Biology 205, no. 1: 85–93. 10.1006/jtbi.2000.2043. 10860702

[ece373810-bib-0026] Longmore, C. , K. Fogarty , F. Neat , et al. 2010. “A Comparison of Otolith Microchemistry and Otolith Shape Analysis for the Study of Spatial Variation in a Deep‐Sea Teleost, *Coryphaenoides rupestris* .” Environmental Biology of Fishes 89, no. 3: 591–605. 10.1007/s10641-010-9674-1.

[ece373810-bib-0027] Mahé, K. , C. Gourtay , G. B. Defruit , et al. 2019. “Do Environmental Conditions (Temperature and Food Composition) Affect Otolith Shape During Fish Early‐Juvenile Phase? An Experimental Approach Applied to European Seabass ( *Dicentrarchus labrax* ).” Journal of Experimental Marine Biology and Ecology 521: 151239. 10.1016/j.jembe.2019.151239.

[ece373810-bib-0028] McKenzie, J. R. 2014. “Review of Productivity Parameters and Stock Assessment Options for Kingfish ( *Seriola lalandi* lalandi).” *New Zealand Fisheries Assessment* Report.

[ece373810-bib-0029] Mille, T. , K. Mahé , M. Cachera , M. C. Villanueva , H. De Pontual , and B. Ernande . 2016. “Diet Is Correlated With Otolith Shape in Marine Fish.” Marine Ecology Progress Series 555: 167–184. 10.3354/meps11784.

[ece373810-bib-0031] MPI . 2002. “Kingfish QMAs, Geospatial Management, Ministry for Primary Industries.” https://data‐mpi.opendata.arcgis.com/datasets/MPI::kingfish‐qmas‐1/about.

[ece373810-bib-0032] Nazir, A. , and M. A. Khan . 2021. “Using Otoliths for Fish Stock Discrimination: Status and Challenges.” Acta Ichthyologica et Piscatoria 51, no. 2: 199–218. 10.3897/aiep.51.64166.

[ece373810-bib-0033] Ndjamba, T. S. I. , M. Araya , and M. E. Oliva . 2022. “Otolith Weight as an Estimator of the Age of *Seriola lalandi* Valenciennes, 1833 (Carangidae), in the Southeastern Pacific.” Animals 12, no. 13: 1640. 10.3390/ani12131640. 35804539 PMC9264935

[ece373810-bib-0034] Neves, A. , A. R. Vieira , V. Sequeira , et al. 2019. “Otolith Shape and Isotopic Ratio Analyses as a Tool to Study *Spondyliosoma cantharus* Population Structure.” Marine Environmental Research 143: 93–100. 10.1016/j.marenvres.2018.11.012. 30477877

[ece373810-bib-0035] Neves, J. , A. Veríssimo , A. Múrias Santos , and S. Garrido . 2023. “Comparing Otolith Shape Descriptors for Population Structure Inferences in a Small Pelagic Fish, the European Sardine *Sardina pilchardus* (Walbaum, 1792).” Journal of Fish Biology 102, no. 5: 1219–1236. 10.1111/jfb.15369. 36880257

[ece373810-bib-0036] Oksanen, J. , F. G. Blanchet , R. Kindt , et al. 2016. “vegan: Community Ecology R Package, Version 2.6‐4. vegan: Community Ecology Package.” R package version 2.4‐1. https://CRAN.R‐project.org/package=vegan.

[ece373810-bib-0037] Parisi‐Baradad, V. , A. Lombarte , E. Garcia‐Ladona , J. Cabestany , J. Piera , and O. Chic . 2005. “Otolith Shape Contour Analysis Using Affine Transformation Invariant Wavelet Transforms and Curvature Scale Space Representation.” Marine and Freshwater Research 56: 795–804. 10.1071/MF04162.

[ece373810-bib-0038] Park, J. M. , M. G. Kang , J. H. Kim , L. A. Jawad , and S. Majeed . 2023. “Otolith Morphology as a Tool for Stock Discrimination of Three Rockfish Species in the East Sea of Korea.” Frontiers in Marine Science 10. 10.3389/fmars.2023.1301178.

[ece373810-bib-0040] Pita, A. , J. Casey , S. J. Hawkins , et al. 2016. “Conceptual and Practical Advances in Fish Stock Delineation.” Fisheries Research 173: 185–193. 10.1016/j.fishres.2015.10.029.

[ece373810-bib-0042] Popper, A. N. , J. Ramcharitar , and S. E. Campana . 2005. “Why Otoliths? Insights From Inner Ear Physiology and Fisheries Biology.” Marine and Freshwater Research 56: 497–504. 10.1071/MF04267.

[ece373810-bib-0043] R Core Team . 2019. “R: A Language and Environment for Statistical Computing.”

[ece373810-bib-0044] Randon, M. , O. Le Pape , B. Ernande , et al. 2020. “Complementarity and Discriminatory Power of Genotype and Otolith Shape in Describing the Fine‐Scale Population Structure of an Exploited Fish, the Common Sole of the Eastern English Channel.” PLoS One 15, no. 11 November: e0241429. 10.1371/journal.pone.0241429. 33151981 PMC7643961

[ece373810-bib-0045] Reig‐Bolaño, R. , P. Marti‐Puig , S. Rodriguez , J. Bajo , V. Parisi‐Baradad , and A. Lombarte . 2010. “Otoliths Identifiers Using Image Contours EFD.” In Advances in Intelligent and Soft Computing, vol. 79, 9–16. Springer. 10.1007/978-3-642-14883-5_29.

[ece373810-bib-0046] Sadighzadeh, Z. , V. M. Tuset , T. Valinassab , M. R. Dadpour , and A. Lombarte . 2012. “Comparison of Different Otolith Shape Descriptors and Morphometrics for the Identification of Closely Related Species of Lutjanus spp. From the Persian Gulf.” Marine Biology Research 8, no. 9: 802–814. 10.1080/17451000.2012.692163.

[ece373810-bib-0047] Schneider, C. A. , W. S. Rasband , and K. W. Eliceiri . 2012. “NIH Image to ImageJ: 25 Years of Image Analysis.” Nature Methods 9, no. 7: 671–675. 10.1038/nmeth.2089. 22930834 PMC5554542

[ece373810-bib-0048] Smith, P. J. , B. Diggles , J. McKenzie , S. Kim , C. Ó~Maolagáin , and P. Notman . 2004. Kingfish Stock Structure: Final Research Report for Ministry of Fisheries Project KIN2002/01, Objective 1 (Tech. Rep.). Ministry of Fisheries.

[ece373810-bib-0049] Vasconcelos, J. , M. Cirera , A. R. Vieira , J. L. Otero‐Ferrer , and V. M. Tuset . 2025. “Application of Shape Analysis for the Identification of Pelagic Fish Stocks.” Hydrobiologia 852, no. 11: 2847–2869. 10.1007/s10750-025-05798-1.

[ece373810-bib-0050] Vignon, M. , and F. Morat . 2010. “Environmental and Genetic Determinant of Otolith Shape Revealed by a Non‐Indigenous Tropical Fish.” Marine Ecology Progress Series 411: 231–241. 10.3354/meps08651.

[ece373810-bib-0051] Waples, R. S. , and O. Gaggiotti . 2006. “What Is a Population? An Empirical Evaluation of Some Genetic Methods for Identifying the Number of Gene Pools and Their Degree of Connectivity.” Molecular Ecology 15, no. 6: 1419–1439. 10.1111/j.1365-294X.2006.02890.x. 16629801

[ece373810-bib-0052] Wickham, H. 2011. “ggplot2.” Wiley Interdisciplinary Reviews: Computational Statistics 3, no. 2: 180–185. 10.1002/wics.147.

[ece373810-bib-0053] Wickham, H. , R. François , L. Henry , and K. Müller . 2020. “A Grammar of Data Manipulation [R Package dplyr Version 1.0.0].” Media.

[ece373810-bib-0054] Wickham, H. , D. Vaughan , and M. Girlich . 2024. “tidyr: Tidy Messy Data.” (Vol. 59) (No. 10).

