# Supplementary material for: Otolith Shape Variation Reveals a Preliminary and Novel Stock Structure Signal of Yellowtail Kingfish ( Seriola lalandi lalandi) in Aotearoa New Zealand
Source: Ecol Evol. 2026 Jul 12;16(7):e73810. doi: 10.1002/ece3.73810 (PMC13358378; doi:10.1002/ece3.73810)
Supplement: Supplementary file 1 — Figure S1: CAP ordination plots for temporal comparisons within sampling regions (Prior Tests 2.1–2.3). Ellipses indicate 95% confidence regions based on multivariate normality. Crosshairs indicate group centroids ±1 SD. Substantial overlap between years in all three regions supports pooling of samples across years for subsequent analyses. Figure S2: Mean deviation from the original outline (%) as a function of the number of Fourier harmonics (left), and the number of wavelet levels (right), used in shape reconstruction. Figure S3: Simulation‐based power analysis for PERMANOVA on standardised wavelet coefficients (four groups: AKW, CEW, CHA, SOU; BPLE excluded; α=0.05; 999 iterations). Power was estimated by bootstrapping from the observed wavelet coefficient distributions across a range of equal sample sizes per group. The red dashed line indicates the 80% power threshold. Dotted vertical lines indicate the actual sample sizes of each sampling area. Three of the four groups—CEW (n=15), AKW (n=27), and CHA (n=33)—meet or exceed the 80% threshold. SOU (n=13) falls marginally below, representing an acknowledged limitation. Table S1: Comparison of left and right otolith shape indices. Normality assessed using the Shapiro–Wilk test. Holm‐adjusted p‐values indicate no significant asymmetry. Table S2: ANCOVA interaction effects of side and fish length on otolith shape indices. Holm‐adjusted p‐values indicate no significant interaction. Table S3: Pairwise Tukey's HSD comparisons of multivariate dispersion (distance to group centroid). Values show difference in dispersion (Diff) and Holm‐adjusted p‐values. Significant values indicate heterogeneity in within‐group variance. [file ECE3-16-e73810-s001.pdf]

Appendices

Appendix - Figures

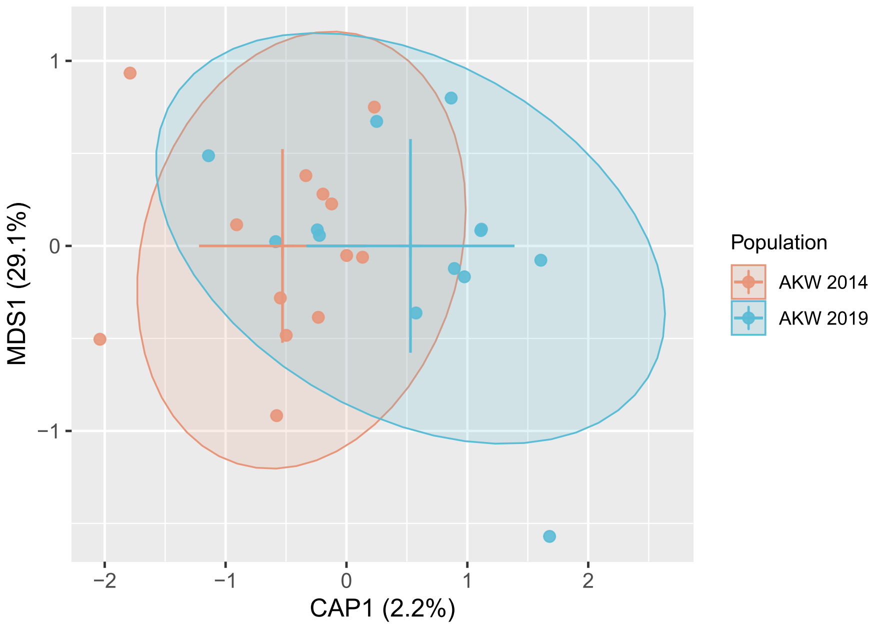

(a) AKW: 2014 vs 2019. PERMANOVA: $F = 0.627$, $R^2 = 0.026$, $p = 0.781$.

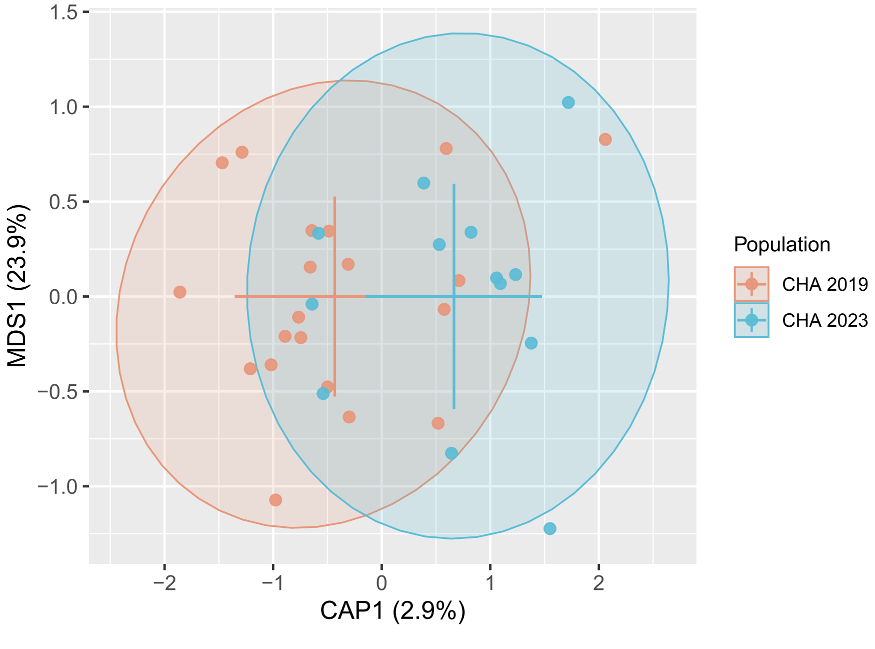

(b) CHA: 2019 vs 2023. PERMANOVA: $F = 0.920$, $R^2 = 0.029$, $p = 0.504$.

Figure S1: CAP ordination plots for temporal comparisons within sampling regions (Prior Tests 2.1–2.3). Ellipses indicate 95% confidence regions based on multivariate normality. Crosshairs indicate group centroids ±1 SD. Substantial overlap between years in all three regions supports pooling of samples across years for subsequent analyses. *(Continued on next page.)*

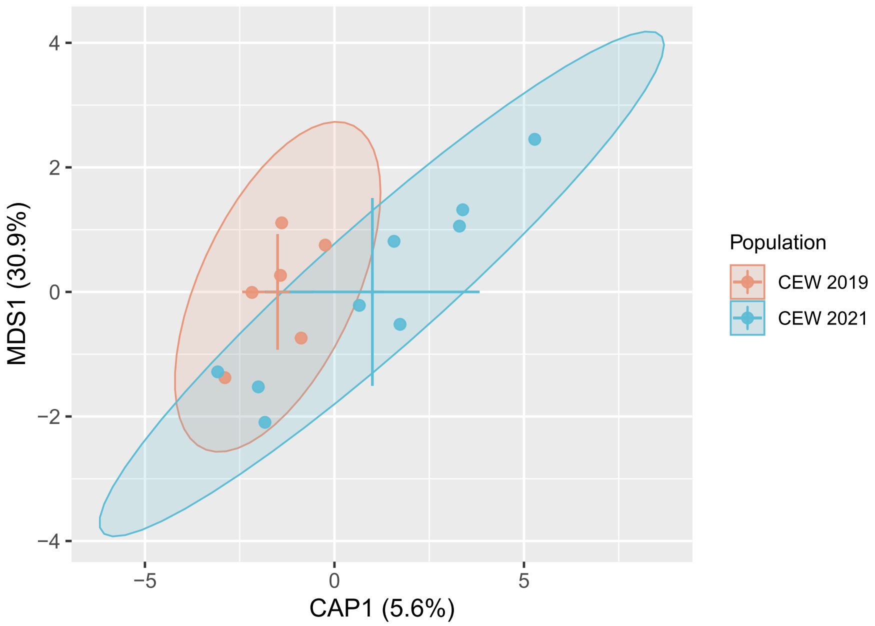

(c) CEW: 2019 vs 2021. PERMANOVA: $F = 0.766$, $R^2 = 0.056$, $p = 0.635$. Note that the 2019 CEW group comprises only six individuals, which limits the power of this comparison.

Figure S1 continued.

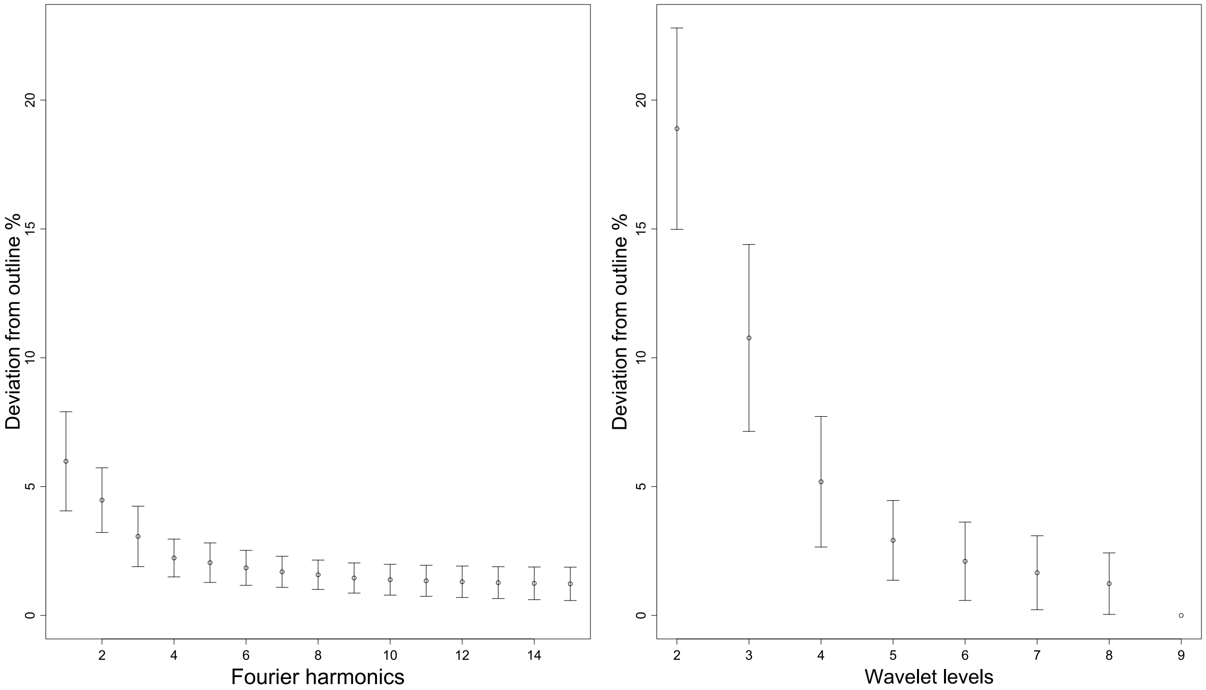

Figure S2: Mean deviation from the original outline (%) as a function of the number of Fourier harmonics (left), and the number of wavelet levels (right), used in shape reconstruction.

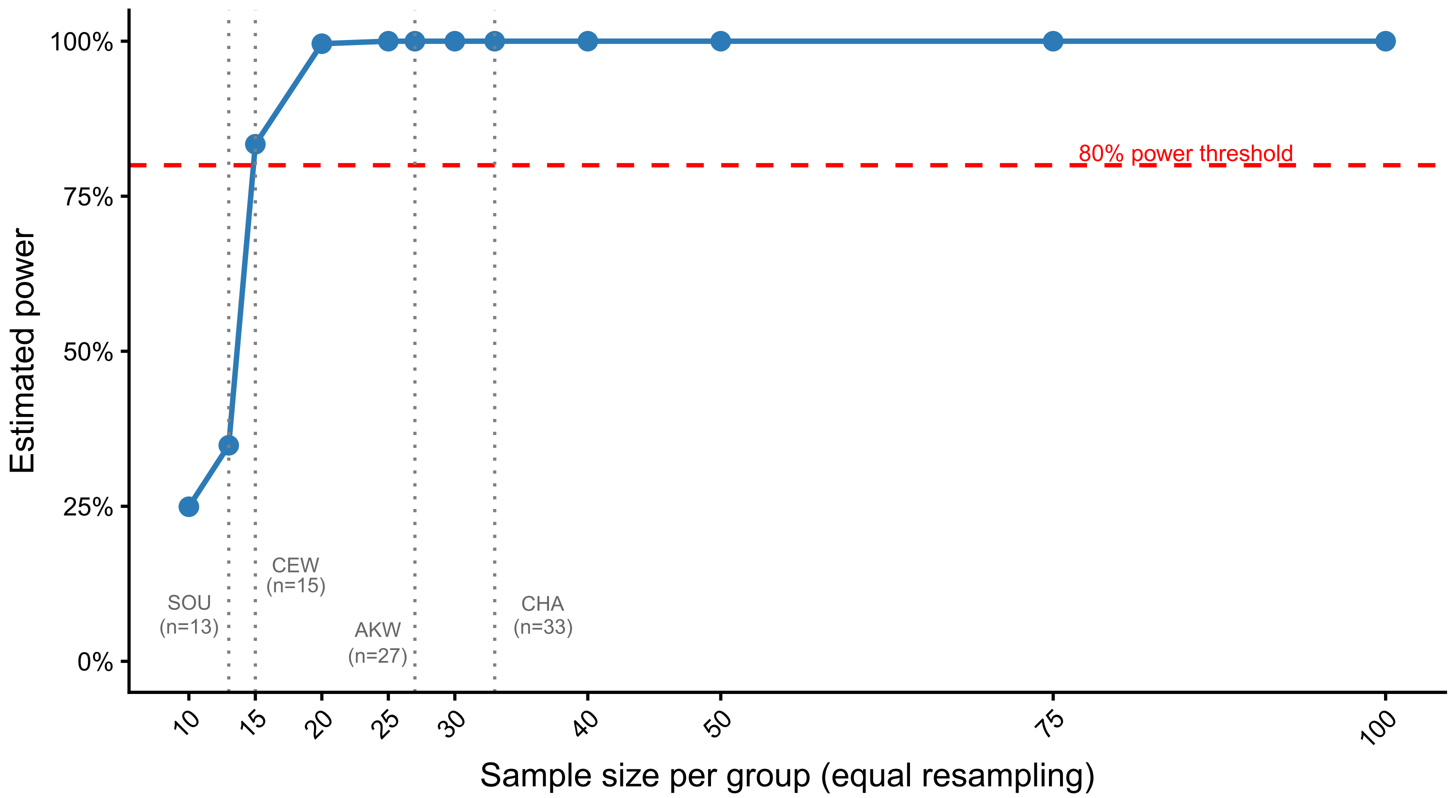

Figure S3: Simulation-based power analysis for PERMANOVA on standardised wavelet coefficients (four groups: AKW, CEW, CHA, SOU; BPLE excluded; $\alpha = 0.05$; 999 iterations). Power was estimated by bootstrapping from the observed wavelet coefficient distributions across a range of equal sample sizes per group. The red dashed line indicates the 80% power threshold. Dotted vertical lines indicate the actual sample sizes of each sampling area. Three of the four groups – CEW ($n = 15$), AKW ($n = 27$), and CHA ($n = 33$) – meet or exceed the 80% threshold. SOU ($n = 13$) falls marginally below, representing an acknowledged limitation.

Appendix - Tables

Table S1: Comparison of left and right otolith shape indices. Normality assessed using the Shapiro-Wilk test. Holm-adjusted p-values indicate no significant asymmetry.

**Shape index**	**Mean left**	**Mean right**	**Shapiro-Wilk** p	**Test type**	**Holm-adjusted** p
Aspect Ratio	2.8418	2.7611	0.6053	Paired t-test	0.1722
Circularity	32.7173	33.1144	0.0188	Wilcoxon signed-rank	0.7904
Ellipticity	0.4774	0.4657	0.8750	Paired t-test	0.1722
Form Factor	0.3890	0.3833	0.0542	Paired t-test	1.0000
Rectangularity	0.5864	0.5853	0.3877	Paired t-test	1.0000
Roundness	0.2644	0.2719	0.3360	Paired t-test	0.3095

Table S2: ANCOVA interaction effects of side and fish length on otolith shape indices. Holm-adjusted p-values indicate no significant interaction.

**Shape index**	**F-value**	p**-value**	**Holm-adjusted** p
Aspect Ratio	0.066	0.7979	1.0000
Circularity	0.005	0.9425	1.0000
Ellipticity	0.085	0.7711	1.0000
Form Factor	0.000	0.9874	1.0000
Rectangularity	0.073	0.7879	1.0000
Roundness	0.004	0.9481	1.0000

Table S3: Pairwise Tukey's HSD comparisons of multivariate dispersion (distance to group centroid). Values show difference in dispersion (Diff) and Holm-adjusted p-values. Significant values indicate heterogeneity in within-group variance.

**Group comparison**	**Diff**	**Holm-adjusted** p	**Significant**
BPLE–AKW	0.572	0.00062	*
CEW–AKW	0.107	0.96167	
CHA–AKW	0.220	0.42287	
SOU–AKW	0.395	0.12787	
CEW–BPLE	-0.465	0.03777	*
CHA–BPLE	-0.352	0.06461	
SOU–BPLE	-0.177	0.83273	
CHA–CEW	0.113	0.94627	
SOU–CEW	0.288	0.53218	
SOU–CHA	0.175	0.81155	
